# Efficacy and Safety of XEN63 Gel Stent Implant over 6 Months for Treatment of Glaucoma

**DOI:** 10.3390/jcm14093036

**Published:** 2025-04-28

**Authors:** Matteo Sacchi, Sara Giammaria, Gloria Roberti, Davide Tomaselli, Gianluca Monsellato, Luca Agnifili, Chiara Posarelli, Giacomo Abbruzzese, Lorenza Ronchi, Filippo Tatti, Stefano Dore, Giuseppe Giannaccare, Paolo Nucci, Antonio Pinna, Michele Figus, Francesco Oddone

**Affiliations:** 1 Department of Medicine, Surgery and Pharmacy, University of Sassari, Viale San Pietro 43, 07100 Sassari, Italy; stefanodore@hotmail.com (S.D.); apinna@uniss.it (A.P.); 2 Ophthalmology Unit, Azienda Ospedaliero-Universitaria di Sassari, 07100 Sassari, Italy; loreronchi@icloud.com; 3 IRCCS Fondazione Bietti, 00198 Rome, Italy; sara.giammaria@fondazionebietti.it (S.G.); gloria.roberti@fondazionebietti.it (G.R.); francesco.oddone@fondazionebietti.it (F.O.); 4 Eye Clinic, San Giuseppe Hospital, IRCCS Multimedica, University of Milan, 20123 Milan, Italy; davide.tomaselli@outlook.com (D.T.); gianluca.monsellato@hotmail.com (G.M.); 5 Ophthalmology Clinic, Department of Medicine and Aging Sciences, University “G. D’Annunzio” of Chieti-Pescara, 66100 Chieti, Italy; luca.agnifili@unich.it; 6 Ophthalmology Unit, Department of Surgery, Medicine, Molecular and Emergency, University of Pisa, 56127 Pisa, Italy; chiara.posarelli@unipi.it (C.P.); figusmichele@gmail.com (M.F.); 7 SS Cosma e Damiano Hospital, 51017 Pescia, Italy; giacomo.abbruzzese@uslcentro.toscana.it; 8 Eye Clinic, Department of Surgical Sciences, University of Cagliari, 09124 Cagliari, Italy; filippotatti@gmail.com (F.T.); giuseppe.giannaccare@gmail.com (G.G.); 9 Department of Clinical Sciences and Community Health, University of Milan, 20122 Milan, Italy; paolo.nucci@unimi.it

**Keywords:** XEN63 gel stent, glaucoma, therapy

## Abstract

**Background/objectives**: The efficacy and safety of the XEN45 gel stent implant in patients with glaucoma have been amply demonstrated. XEN63 is a new device that has been developed with a larger bore. This multicenter, observational, retrospective study assessed the efficacy and safety of XEN63 in patients with glaucoma. **Methods**: Medical records from six participating centers were screened to identify patients meeting the inclusion criteria. The primary outcome was mean IOP at 6 months after surgery. **Results**: The study included 114 eyes from 102 patients (XEN63 alone: 68 eyes, and XEN63 + Phaco: 46 eyes); 92% of patients had primary open-angle glaucoma. Baseline IOP for all patients was a median of 23.0 mmHg (IQR: 18.5–27.5 mmHg), which decreased significantly on day one post-surgery to 7.0 mmHg (IQR: 4.5–9.5 mmHg) and gradually stabilized at around 13.5 mmHg (IQR: 10.5–16.5 mmHg) by 6 months with no significant differences between groups at 6 months. The number of ocular hypertensive medications (OHMs) reduced significantly from a baseline median of 2.7 ± 1.1 to 0.5 ± 1.0 at 6 months in the entire cohort. The XEN63 alone group showed a significantly lower need for OHMs at 3 and 6 months. The surgical success rate was comparable between the two groups (54.4% vs. 47.8%, *p* = 0.05, XEN63 alone and XEN63 + Phaco). There was no statistically significant difference in survival outcomes between the XEN63 (0.59, 95% CI: 0.49–0.73) and XEN63 + Phaco groups (0.55, 95% CI: 0.42–0.72) (*p* = 0.89). **Conclusions**: In the largest study with XEN63 to date, the device appears to significantly decrease the IOP and the OHMs. Simultaneous XEN63 implant and phacoemulsification showed similar outcomes compared to XEN63 alone.

## 1. Introduction

Glaucoma refers to a broad range of chronic, multifactorial, and progressive optic neuropathies characterized by progressive loss of ganglion cells in the retina with consequent defects in the visual field [1]. The condition remains a primary cause of irreversible blindness [2]. The main aim of treatment is to slow disease progression and improve the quality of life, which can be achieved by decreasing intraocular pressure (IOP) [3]. In order to achieve this, a number of different strategies, including topical medications, laser treatment, and surgery, are currently available [3]. While topical hypotensive medication is often the initial treatment, many patients will not achieve adequate control of glaucoma due to side effects, poor adherence, and lack of efficacy [4,5].

Trabeculectomy is widely considered the gold standard for surgical treatment of glaucoma and has well-established efficacy in lowering IOP, with nearly 90% of patients having qualified success after 20 years [6]. To minimize the invasiveness of surgery, minimally invasive glaucoma surgery devices have been developed with the aim of achieving a safer and less traumatic way of lowering IOP [7]. Among the different devices, the gel stent implant XEN^®^ has been developed, which allows aqueous humor to flow from the anterior chamber to the subconjunctival space [8]. A large number of studies have demonstrated the good efficacy and safety profile of XEN45 in patients with open-angle and other types of glaucoma. In a systematic review and meta-analysis of 14 studies on 963 eyes, IOP was found to be significantly lowered by a mean of 7.4 mmHg at 24 months, with a mean reduction of 1.7 medications [9]. A registry-based prospective study reported an overall success of 68% at three years of follow-up, with a significant decrease of ocular hypotensive medications (OHMs) from 2.7 ± 0.9 to 0.9 ± 1.1 (*p* < 0.01) [10]. In addition, a real-world study over 2 years reported that the success rate with the XEN45 device was 66% and that IOP was decreased by 44%, while reducing the number of medications by two per day [11].

Following the promising results with the XEN45 device, a new device, namely XEN63, was developed [12]. The main difference between the two devices is the bore of the stent, which is 45 μm in XEN45 compared to 63 μm in XEN63 [12]. In addition, since the outer diameter of XEN63 is greater than that of XEN45 (0.25 mm vs. 0.22 mm), the side flow with the former is reduced compared to the latter. Both devices are introduced with a 27 G needle. The new device was developed in order to decrease the incision site and increase the flow of aqueous humor with a larger bore size. Compared to XEN45, the clinical evidence for XEN63 is more limited. In a 12-month study in primary open-angle glaucoma (POAG) in 80 eyes from 80 patients, XEN63, alone or in combination with phacoemulsification, significantly lowered IOP by around 20 mmHg and decreased the number of ocular hypotensive medications to about 0.7/0.8 [13]. A pilot study in six patients with POAG also reported that five cases had complete success after 2 years [14].

Given the limited evidence for the XEN63 gel stent implant in the treatment of glaucoma, we carried out a retrospective, multicenter study to assess the efficacy and safety of the device in terms of lowering IOP and reducing the number of ocular hypotensive drugs in 114 eyes from 102 patients. Here, we report the short-term results of our analysis.

## 2. Materials and Methods

### 2.1. Study Design

This was a multicenter, observational, retrospective study of patients with glaucoma who underwent XEN63 gel stent implantation (AbbVie, North Chicago, IL, USA) between 1 April 2023 and 1 April 2024 across 6 Italian centers (Eye Clinic, Azienda Ospedaliera Universitaria, University of Sassari; University Eye Clinic San Giuseppe, IRCCS Multimedica, Milan; IRCSS Bietti Rome; Ophthalmology Clinic, Department of Medicine and Ageing Science, University “G. D’Annunzio” of Chieti-Pescara; Eye Clinic, SS Cosma e Damiano Hospital, Pescia, Italy; Eye Clinic, Department of Surgical Sciences, University of Cagliari, Cagliari). All participating sites were trained in the study protocol and in the requirements for anonymized data entry. Prior to study initiation, the study protocol received approval from an independent ethics committee at each site, in accordance with local regulations. The study adhered to the principles of the Declaration of Helsinki, as well as European and national data protection laws and local regulations in effect at the time.

### 2.2. Study Implant and Surgical Technique

The XEN^®^ 63 gel stent implantation (AbbVie, North Chicago, IL, USA) is a 6 mm long, cross-linked porcine collagen stent with a 63 μm inner lumen diameter, preloaded into a single-use injector. In the combo group, subjects underwent corneal phacoemulsification with intraocular lens implantation before implantation of the gel stent, and if needed, acetylcholine was injected into the anterior chamber (AC) before insertion. Intraoperative 0.02% mitomycin C (MMC; 0.1 mL) was injected sub-conjunctively under the tenon capsule with a 30 G needle and then spread with a microsponge applied to the conjunctiva in the superior nasal quadrant where the implant was to be inserted. Using an ab interno approach, the preloaded injector needle was inserted through a 1.8 mm corneal incision for solo procedures or a 2.2 mm incision for combo procedures, with the AC filled with viscoelastic material. The needle was directed across the AC to the superonasal quadrant. A goniolens could be used to verify placement and avoid trauma. The implant was positioned 3.0 mm posterior to the limbus, exiting through the sclera into the subconjunctival space, with 1 to 2 mm left in the AC. Viscoelastic material was removed from the AC by irrigation/aspiration in the XEN63 + Phaco group or by saline washing in the XEN63 group. Topical OHMs, as well as systemic IOP-lowering medications, were suspended from the day of surgery, as is common practice. Post-operative needlings were performed with MMC or 5-fluorouracil at the discretion of the surgeon.

### 2.3. Patient Identification and Data Extraction

Medical records from each site were screened to identify consecutive patients meeting the following criteria: (1) age ≥ 18 years; (2) affected by glaucoma (including all forms of open-angle glaucoma and narrow-angle glaucoma if the irido-corneal angle was deemed accessible for the procedure) or ocular hypertension; (3) who underwent ab interno implantation of the XEN^®^ 63 gel stent as a standalone procedure (XEN63 group) or in combination with cataract surgery (XEN63 + Phaco group) between 1 April 2023 and 1 April 2024; (4) who had a minimum required data set at baseline including age, gender, ethnicity, IOP, number of OHMs, type of glaucoma, and lens status; (5) who had the minimum required data during follow-up in at least 5 of 6 time points (1 day, 1 week, 1 month, 2 months, 3 months, 6 months) for IOP and OHMs. Patients not meeting all 5 criteria were not included. If two eyes from the same patient were eligible, both were recruited. Each eye selected was assigned a unique identifier, and data were extracted from the patient’s medical record, starting at the baseline visit through the date of the last follow-up at 6 months from surgery. Patients with previous glaucoma surgery could be included.

Glaucoma was diagnosed according to the European Glaucoma Society Guidelines [15]. Patients who underwent surgery met at least one of the following criteria: (1) unmet target IOP despite maximally tolerated topical medications (including oral acetazolamide), (2) intolerance to glaucoma therapy, and (3) significant glaucoma progression confirmed on three consecutive reliable visual fields (VF) (30-2 test, full-threshold) (Humphrey field analyzer II 750; Carl Zeiss Meditec Inc., Dublin, CA, USA).

### 2.4. Outcomes

The primary outcome was mean IOP at 6 months after surgery. Secondary outcome measures included percentage of IOP reduction, number of OHMs from baseline to 6 months at all time points (1 day, 1 week, 1 month, 2 months, 3 months, 6 months), percentage of eyes achieving surgical success, incidence of additional procedures, and adverse events. “Success” was defined as (1) final IOP ≤ 17 mmHg, (2) IOP reduction ≥ 20% from preoperative value, and (3) absence of the following conditions: IOP > 17% for two consecutive visits after one month of follow-up; need for surgical revisions or reoperation; clinically significant macular hypotony (IOP < 6 mmHg and loss of 2 lines of visual acuity). Eyes not meeting all the 3 criteria were considered as “failure”. “Complete success” was defined as the satisfaction of the previous criteria with no OHMs, while “qualified success” was defined as the satisfaction of the criteria with the help of OHMs. Needlings (at any time) and IOP > 17 mmHg in the first month of follow-up were not considered as failure criteria.

### 2.5. Statistical Analyses

Descriptive statistics were summarized using median and interquartile range (IQR) for continuous variables, and frequency (number and percentage) for categorical variables. Normality of distributions was assessed using the Shapiro–Wilk test. Continuous variables were compared using either the 2-sided Student’s *t*-test or the Mann–Whitney U test, depending on the distribution of the data. Categorical variables were analyzed using Chi-square or Fisher’s exact tests, as appropriate. To evaluate within-group changes in IOP and the number of OHMs, repeated measures ANOVA with Greenhouse–Geisser correction was employed. The issue of missing data was handled by performing both per-protocol (PP) and last observation carried forward (LOCF) analyses. Kaplan–Meier survival curves were used to determine the cumulative probability of surgical success. In survival analysis, patients were censored if their follow-up concluded before meeting the failure criteria. Clustered survival models were applied to account for inter-eye correlation. Univariate and multivariate Conditional Cox proportional hazards models were employed to examine the association between preoperative factors and surgical failure. The validity of the proportional hazards assumptions was assessed using the Martingale residuals method. Statistical significance was determined at a *p*-value of <0.05. Statistical analysis was conducted using the open-source software R (version 3.6.0) with the survival (version 3.2-7) and survminer (version 0.4.9) packages.

## 3. Results

### 3.1. Patient Characteristics

The study included 114 eyes from 102 patients, with 68 eyes in the XEN63 group and 46 eyes in the XEN63 + Phaco group (Table 1). The median age of patients was 74 years (IQR: 65–80), with no significant differences between the two groups in terms of age distribution (*p* = 0.87). The majority of patients were Caucasian (98%), with small representations from Afro-American (n = 1) and Asian (n = 1) ethnicities. Regarding the type of glaucoma, the majority of patients (92%) had primary open-angle glaucoma (POAG), with similar distributions of other types (PXF, PACG, and NVG) between the two groups (*p* = 0.3). Overall, no significant differences were reported on previously performed surgeries/lasers (*p* = 0.2). In terms of lens status, 32.3% of eyes in the XEN63 group were phakic at baseline, while all eyes in the XEN63 + Phaco group were phakic due to the planned cataract surgery. No significant differences were observed between groups for VF damage, C/D Ratio, and global RNFL (VF damage *p* = 0.3; C/D ratio *p* = 0.04; global RNFL *p* = 0.6). Patients in the XEN63 group had a higher preoperative IOP (<0.001). Baseline endothelial cell count was available for 32 eyes. The XEN63 + Phaco group had a slightly higher baseline endothelial cell count (2240.4 ± 404.4 cells/mm^2^) compared to the XEN63 group (2059.1 ± 265.5 cells/mm^2^), but the difference was not statistically significant (*p* = 0.22).

### 3.2. Clinical Outcomes

**Figure 1:**
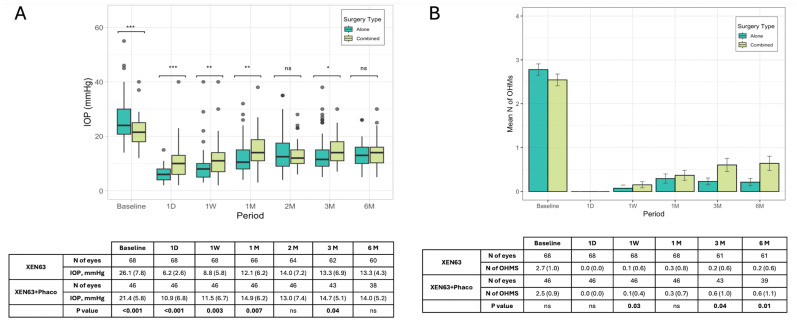
Changes in IOP (**A**) and number of OHMs (**B**) over 6 months in XEN 63 and XEN63 + Phaco groups. ns: >0.05; *: <0.05; **: <0.01; ***: <0.001.

Considering the primary outcome, baseline IOP for all patients was a median of 23.0 mmHg (IQR: 18.5–27.5; mean: 24.2; SD: 7.4 mmHg), which decreased significantly on day one post-surgery to 7.0 mmHg (IQR: 4.5–9.5; mean: 8.1; SD: 5.3 mmHg) and gradually stabilized at around 13.5 mmHg (IQR: 10.5–16.5; mean: 13.5; SD: 4.7 mmHg) by 6 months. Differences in outcomes between patients treated with XEN63 alone versus XEN63 combined with phacoemulsification (XEN63 + Phaco) were also analyzed. The XEN63 group (n = 68) showed a slightly higher baseline IOP of 24.0 mmHg (IQR: 19.3–28.6; mean: 26.1; SD: 7.8 mmHg) compared to XEN63 + Phaco (median: 21.5, IQR: 18.0–25.0; mean: 21.4; SD: 5.8 mmHg, *p* < 0.001), which reduced to 13.3 mmHg (IQR: 10.0–6.0; mean: 13.3, SD: 4.3 mmHg) at 6 months. The XEN63 + Phaco group (n = 46) started with a slightly lower baseline IOP of 21.5 mmHg (IQR: 18.0–25.0 mmHg) and reached a similar value of 14.0 mmHg (IQR: 10.2–16.0, mean: 14.0, SD: 5.2 mmHg) at 6 months. Statistical comparisons (Wilcoxon test) showed a significantly lower value of IOP in the XEN63 alone group during the first month of follow-up, but no significant difference at the end of the follow-up (Figure 1A).

The LOCF analysis showed a similar result compared to the PP analysis for both the entire sample and the subgroup analysis.

**Figure 2:**
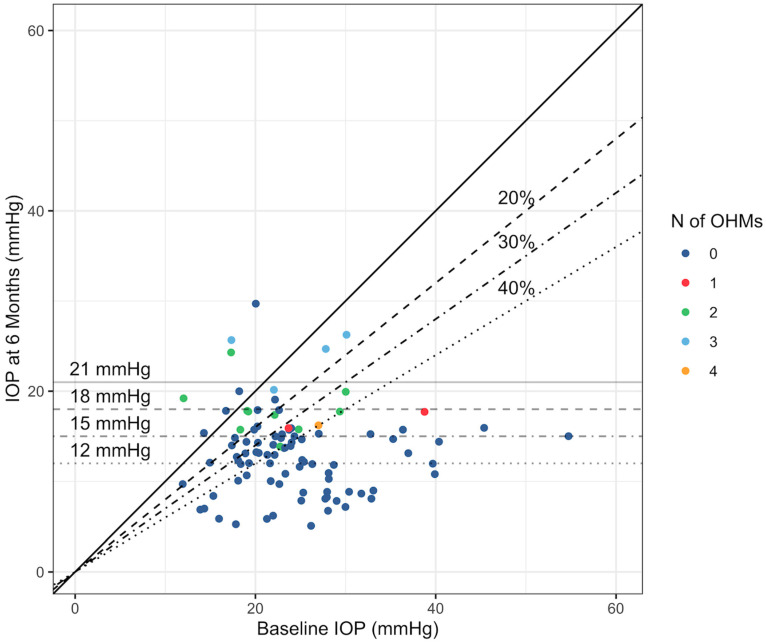
Trends in the number of OHMs needed at 6 months by baseline IOP.

The secondary outcomes of the study focused on changes in IOP across different baseline IOP subgroups and achievement of specific reduction thresholds and absolute levels. Patients with higher baseline IOP experienced more significant reductions, with the ≥36 mmHg group showing a median IOP reduction of −64.9%, compared to −20.0% in the ≤15 mmHg group. The scatterplot in Figure 2 shows the trends in the number of OHMs needed at 6 months by baseline IOP and IOP at 6 months.

The majority of patients achieved significant results: 72.8% reached at least a 15% reduction, 70.1% achieved a 20% reduction, 64.0% reached a 30% reduction, and 44.7% saw a reduction of ≥40%. Regarding absolute IOP targets, 81.5% of patients reached an IOP of ≤21 mmHg at 6 months, and 35.9% achieved an IOP ≤ 12 mmHg. The difference in IOP reduction between the groups was not statistically significant.

The number of OHMs reduced significantly from a baseline median of 2.7 ± 1.1 to 0.5 ± 1.0 at 6 months in the entire cohort. Both the XEN63 and XEN63 + Phaco groups showed similar trends in reducing the need for medications, with a lesser need for medical treatment in the XEN63 group vs. the XEN63 + Phaco at 3 (0.2 ± 0.6 vs. 0.6 ± 1.0, *p* = 0.04) and 6 (0.2 ± 0.7 vs. 0.6 ± 1.1, *p* = 0.01) months (Figure 1B). The PP analysis did not show comparable figures to the LOCF analysis in either the entire sample or in the two groups.

**Figure 3:**
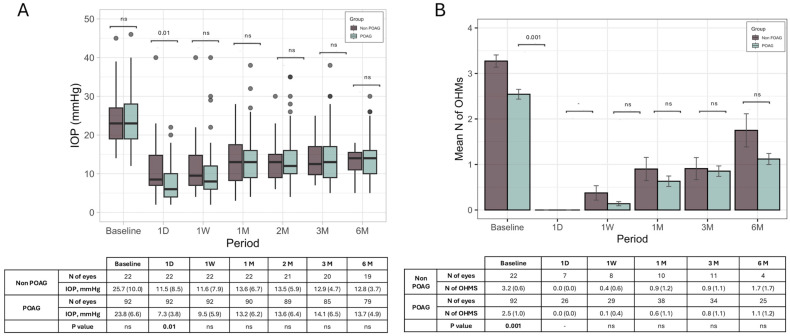
Changes in IOP (**A**) and number of OHMs (**B**) over 6 months in non-POAG and POAG groups. ns: >0.05.

An additional analysis of changes in IOP and the OHMs in POAG cases did not show any significant difference compared to non-POAG cases (Figure 3).

### 3.3. Surgical Outcomes

Considering the definition of success, overall, 59 eyes (51.7%) met success criteria, with the majority (94.9%) achieving complete success. XEN63 alone achieved a higher success rate (54.4%) compared to XEN63 + Phaco (47.8%), although the difference was not statistically significant (*p* = 0.05). The failure rate across all patients was 38.6%, with 26 eyes (38.2%) in the XEN63 group and 18 eyes (39.1%) in the XEN63 + Phaco group (*p* = 0.34).

Failed IOP reduction (both final IOP ≤ 17 mmHg and final IOP reduction ≥ 20% not reached) accounted for 35.1% of failures across all patients, with different rates between groups. Among patients who received only XEN63, 23.0% experienced failed IOP reduction, compared to 41.1% in the XEN63 + Phaco group. The failure rate for the criteria final IOP ≥18 mmHg and IOP reduction ≥ 20% was 16.2% and 10.8%, respectively. The first criterion led to failure in 19.2% of cases in the XEN63 group and in 5.8% of cases in the XEN63 + Phaco group, while the second criterion led to failure in 3.8% of cases in the XEN63 group and in 17.6% of cases in the XEN63 + Phaco group. Consecutive IOP > 17 mmHg and IOP spikes accounted for 37.8% (30.7% vs. 35.3%) and 16.2% (19.2% vs. 11.1%) of failures across all patients. Only one case of hypotony maculopathy was registered in the XEN63 group. Eleven eyes (9.6%) ended the follow-up before 6 months. The LOCF analysis, which also included subjects lost during follow-up, did not show a result comparable to those of the PP analysis (XEN63 success rate 60.3%, XEN63 + Phaco success rate 52.1%).

**Figure 4:**
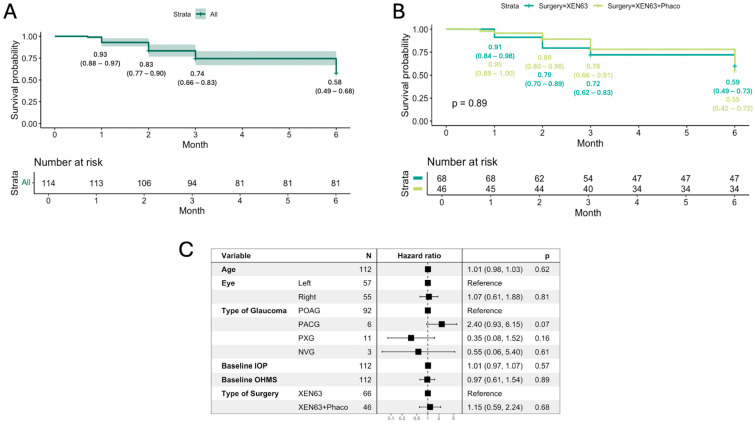
Kaplan–Meier survival probability for all patients (**A**) and for the XEN63 and XEN63 + Phaco groups (**B**). Forest plot showing hazard ratio for failure considering different factors (**C**).

At the end of follow-up, the Kaplan–Meier survival probability for all patients was 0.58 (95% CI: 0.49–0.68) (Figure 4A). There was no statistically significant difference in survival outcomes between the XEN63 (0.59, 95% CI: 0.49–0.73) and XEN63 + Phaco groups (0.55, 95% CI: 0.42–0.72) (*p* = 0.89) (Figure 4B).

Univariate and multivariate Cox regression analysis showed no significant associations between preoperative factors such as age, laterality, type of glaucoma, baseline IOP, and OHMs with the risk of surgery failure (all parameters, *p* > 0.05). Additionally, the type of surgery (XEN63 vs. XEN63 + Phaco) did not significantly influence the hazard ratio for failure (HR = 1.1, *p* = 0.84) (Figure 4C).

**Figure 5:**
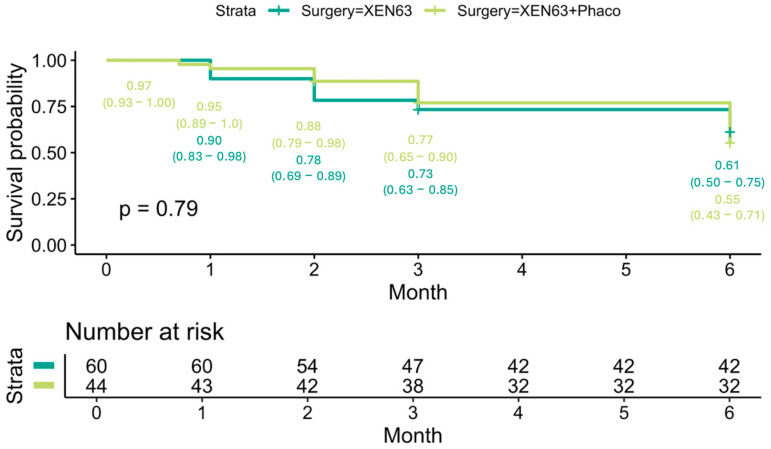
Kaplan–Meier survival probability of patients with open-angle (OH, POAG, PXF) for the XEN63 and XEN63 + Phaco groups.

As we included 10 cases diagnosed with non-open-angle glaucoma (six PAC, three neovascular, and one uveitic glaucoma), we performed a separate analysis excluding these cases. The open-angle group (104 eyes) showed a Kaplan–Meier survival probability comparable to that of the overall group (open-angle + non-open-angle, 114 eyes), with survival probabilities of 0.59 (95% CI: 0.50–0.70) and 0.58 (95% CI: 0.49–0.68), respectively (*p* = 0.93) (Figure 5).

### 3.4. Safety

The complications observed are reported in Table 2. Complications were observed in 46 eyes (40.3%) during the follow-up period. Early complications (≤1 month of follow-up) included hyphema, choroidal detachment, and bleb fibrosis. Among late complications (≥3 months of follow-up), bleb fibrosis was more common after 3 months. Hypotony maculopathy occurred in three eyes, one of which was clinically significant (in the XEN63 group), whereas the others were asymptomatic. Five eyes experienced IOP > 30 mmHg, which required at least one additional surgical procedure.

A total of 49 eyes (42%) underwent at least one additional post-operative intervention during follow-up, with 35 interventions performed in the XEN63 group and 42 interventions performed in the XEN63 + Phaco group (Table 3). Needling procedures were the most common intervention and were performed 19 times in the XEN63 group and 34 times in the XEN63 + Phaco group, with a predominance for needling without the use of antimetabolites. In augmented needlings, MMC was injected at the end of the procedure. In both groups, surgical revision was conducted four times. Reoperations (Phaco + Trabeculectomy, Trabeculectomy, PreserFlo^TM^ MicroShunt implantation, Xen^®^ Gel Stent ab externo implantation) were performed 10 times in the XEN63 Group and 2 times in the XEN63 + Phaco group.

At 6 months, BCVA was stable compared to baseline in the XEN63 group (*p* = 0.99) but improved significantly in the XEN63 + Phaco group (*p* = 0.02), which is likely attributable to the additional cataract surgery in this group.

The endothelial cell count showed a statistically significant decrease of 11.8% at 6 months (*p* = 0.002). The XEN63 + Phaco group experienced a slightly higher reduction in endothelial cell density (−12.6%) compared to XEN63 alone (−10.3%), although the difference was not statistically significant (*p* = 0.14).

### 3.5. Statistical Power

A post-hoc power analysis was performed for an alpha level of 0.05 to check the significance of the results obtained for the reduction of IOP and number of OHMs, considering the whole sample. The power obtained was >0.99 for both variables.

## 4. Discussion

The present study assessed outcomes and safety in 114 eyes from 102 patients with glaucoma who received the XEN63 gel stent implant either alone or in combination with phacoemulsification. In the entire cohort, the primary outcome measure of IOP decreased from a median of 23.0 mmHg to 8.1 mmHg on the day after surgery, and gradually stabilized to 13.5 mmHg after 6 months. Improvements in IOP were also very similar in patients receiving or not receiving concomitant phacoemulsification. Likewise, the number of OHMs was reduced significantly from a median of 2.7 ± 1.1 to 0.5 ± 1.0 at 6 months in the entire cohort, with similar trends in both groups. The decreases in IOP observed with XEN63 are broadly comparable to those seen with XEN45. In a large meta-analysis of 14 studies on 963 eyes, the mean decrease in IOP was 7.44 mmHg at 24 months compared to a decrease of 9.5 mmHg at 6 months herein [9]. While the timepoints are not comparable, long-term studies have shown that outcomes at early timepoints are largely maintained for up to 3 and 5 years [16,17]. In the above-mentioned meta-analysis, there was also a reduction in medications by 1.67 at 24 months, similar to that observed herein [9]. Accordingly, the mean reduction in IOP observed with XEN63 herein is similar to those consistently seen with XEN45. Patients in the XEN63 alone group had a higher baseline IOP compared to the XEN63 + Phaco group. Although a higher baseline IOP could have been driven by a less favorable outcome, the final mean IOP and surgical survival rate were comparable between the two groups, and the number of antiglaucoma medications was lower in the XEN63 alone, meaning that the baseline IOP is not a predictor of clinical success. This is in agreement with the univariate and multivariate Cox regression analysis we made in this study and with the results of a previous work of our group [10].

Notably, although patients in the XEN63 + Phaco group had a lower baseline IOP, they required a slightly but significantly higher number of medications to achieve similar IOP levels at 6 months compared to the XEN63 alone group. The post-operative inflammation induced by phacoemulsification may reduce the surgical success of combined procedures, as previously demonstrated in phaco-trabeculetomy [18].

Further studies with longer follow-up are warranted to clarify whether a staged approach might be preferable when both glaucoma and cataract surgery are indicated.

There are many fewer clinical studies with XEN63 than with XEN45. In a 3-month analysis of 23 eyes in 23 patients, mean IOP significantly decreased from 27.0 mmHg at baseline to 12.2 mmHg at study end [12]. In that analysis, 61% and 70% of eyes achieved an IOP reduction of ≥30% and ≥20%, respectively, while in our study, 70.1% achieved a 20% reduction and 64.0% reached a 30% reduction. The number of OHMs also decreased from 2.3 drugs to 0.1 drugs at 3 months. In a follow-up analysis of the same cohort, the mean reduction in IOP was 14.1 mmHg, and the mean number of OHMs was 1.0 after 18 months [19].

A study by Martínez-de-la-Casa et al. in 80 eyes in 80 patients with POAG reported that XEN63 implantation was associated with significant lowering of preoperative IOP which was also significantly lowered at 12 months from 22.1 mmHg to 14.7  mmHg receiving the XEN63 standalone and from 19.8 mmHg to 13.8 mmHg in those receiving XEN63  +  phacoemulsification with no significant differences between groups [13]. OHMs were also reduced from 2.5 to 0.3 drugs in the standalone group and from 2.0 to 0.3 drugs in the combination group. Thus, implantation of XEN63 appears to be associated with similar efficacy whether or not the procedure is combined with phacoemulsification.

The endothelial cell counts in our cohort decreased by 11.8% from baseline at 6 months, with no significant differences between groups. For XEN45, endothelial loss appears to be low after implantation, even after 5 years of follow-up [20]. Moreover, no differences were seen with XEN45 regarding the position of the device and endothelial cell loss. This would suggest that neither XEN45 nor XEN63 has any substantial impact on the loss of endothelial cell counts.

The safety profile of XEN45 is well characterized and very favorable. In a meta-analysis of 33 studies in 3062 eyes receiving XEN45 implantation for POAG, hypotony was the most common post-operative complication, and was seen in 20% of patients [21]. Of note, most cases of hypotony were numerical hypotony and did not require intervention. In terms of frequency, this was followed by gross hyphema (14%), transient IOP spikes > 30 mmHg (13%), stent exposure (1%), and stent migration (1%). Hyphema was also a common early post-operative complication in our cohort of eyes (four cases on day 1 and one case within 1 week). Intraocular hypertension was seen in a total of three cases (at 1 week, 3 months, and 6 months). Dislocation and malposition occurred in one case each, both at 3 months. In contrast, bleb fibrosis was a relatively common event occurring late in our series, with two and six cases seen at 3 and 6 months, respectively.

In our cohort, needling procedures were the most common post-operative intervention (47% of patients) and were required in 19 eyes in the XEN63 group (28%) and in 34 eyes in the combination group (74%), with most procedures performed without the use of antimetabolites. In the meta-analysis by Lim et al. on XEN45 standalone implantation, 38% of eyes required at least one post-operative needling [9]. Similarly, in the XEN–Glaucoma Treatment Registry on 239 eyes, needling was needed in 23.0% of eyes at least once during follow-up of at least 1 year [8]. Needling after XEN implantation can be performed with or without the use of antimetabolites. In the current study, approximately 60% of needling procedures were carried out without antimetabolites. In previous studies, the proportion of non-augmented needlings ranged from 20% [17] to 50% [10]. Antimetabolites such as mitomycin C (MMC) can increase the success rate of the procedure, but are also associated with risks such as device exposure and blebitis. The decision to use antimetabolites during needling should be based on bleb morphology, the degree of inflammation, and the time elapsed since implantation. Early mechanical obstruction of the XEN in a non-inflamed bleb can often be managed with non-augmented needling, whereas MMC should be considered in cases with signs of ongoing bleb fibrosis.

Among the limitations of the present study are the relatively short follow-up time of 6 months, and longer follow-up is needed to better understand the long-term efficacy and safety of the device. Notwithstanding, as mentioned, most complications occur in the early post-operative phase, and the efficacy at longer follow-up is similar to that seen at earlier times [16,17]. Thus, the results at shorter times appear to be indicative of long-term outcomes. In addition, some interventions were performed by surgeons who were still on the learning curve of the procedure, and thus, outcomes and potential complications may not necessarily reflect those achieved by surgeons with vastly more experience. Indeed, it has been reported that intraoperative difficulties are variable and related to the learning curve [8].

Another limitation of our study is that a small proportion of patients had both eyes included in the analysis. In addition, a few patients in the XEN63 alone group had a diagnosis of PACG. A possible explanation is that during the preoperative assessment, the surgeon evaluated the angle and determined it to be sufficiently open for implantation, even in a patient with PACG. Although it is possible, we believe that an XEN implant should be cautiously implanted in patients with narrow angles due to the risk of corneal endothelium and iris contact. Cataract removal has a limited effect in patients with POAG, but can significantly improve IOP control in those with PACG. Although we included a small proportion of patients with PACG, only two patients were in the XEN63 + Phaco group. We believe that the inclusion of this small percentage of patients should not bias the overall results of our analysis. Nonetheless, this does mirror a real-world setting.

In conclusion, to the authors’ knowledge, this is the largest study with XEN63 carried out to date. Overall, in terms of efficacy and post-operative complications and procedures, XEN63 can be considered safe and effective in lowering IOP and reducing the need for OHMs and is a valid surgical option in patients with glaucoma failing less invasive therapies. Further comparative studies will elucidate the differences in clinical outcome and safety between XEN63 and other filtering surgeries, including XEN45.

## Figures and Tables

**Table 1:** Demographic and ocular characteristics.

Demographic Characteristics				
	All Patients (n = 114)	XEN63 Alone (n = 68)	XEN63 + Phaco (n = 46)	*p*-Value
**Age, median (IQR)**	74.0 (65.0; 80.0)	75.5 (64.7; 81.0)	72.5 (65.5; 79.2)	0.7
**Ethnicity**				0.3
Caucasian	100 (98.0%)	67 (98.5%)	45 (97.8%)	
Afro-American	1 (0.9%)	0 (0.0%)	1 (2.2%)	
Asian	1 (0.9%)	1 (1.5%)	0 (0.0%)	
**Systemic conditions**	**(n = 59)**	**(n = 28)**	**(n = 31)**	0.6
None	8 (18.6%)	5 (17.8%)	3 (9.6%)	
Diabetes	10 (%)	4 (14.2%)	6 (19.3%)	
Hypertension	42 (%)	20 (71.4%)	22 (70.9%)	
Hypercholesterolemia	13 (%)	6 (21.4%)	7 (22.5%)	
Cardiovascular diseases	8 (%)	3 (10.7%)	5 (16.1%)	
Thyroid Dysfunction	6 (%)	4 (14.2%)	2 (6.4%)	
Prostatic Hypertrophy	3 (%)	2 (7.1%)	1 (3.2%)	
Parkinson	1 (%)	0 (0.0%)	1 (3.2%)	
Alzheimer	1 (%)	1 (3.5%)	0 (0.0%)	
Pulmonary diseases	3 (%)	1 (3.5%)	2 (6.4%)	
Depression–Anxiety	4 (%)	1 (3.5%)	3 (9.6%)	
Autoimmune diseases	5 (%)	2 (7.1%)	3 (9.6%)	
**Ocular Characteristics**				
	**All Patients** **(n = 114)**	**XEN Alone** **(n = 68)**	**XEN + Phaco** **(n = 46)**	***p*-Value**
**BCVA, median (IQR) (n = 69)**	0.6	0.6	0.6	0.2
	(0.4–1.0)	(0.3–0.9)	(0.5–1.0)	
**IOP, median (IQR)**	23.0	24	21.5	<0.001
	(19.0–28.0)	(20.7–30.0)	(18.0–25.0)	
**N of Drugs, median (IQR)**	3 (2–3)	3 (2–4)	3 (2–4)	0.2
**Type of Glaucoma**				0.3
POAG	92 (80.7%)	55 (80.8%)	37 (80.4%)	
PXF	11 (9.6%)	4 (5.8%)	7 (15.2%)	
PACG	6 (5.2%)	4 (5.8%)	2 (4.3%)	
OHT	1 (0.8%)	1 (1.4%)	0 (0.0%)	
NVG	3 (2.6%)	3 (4.4%)	0 (0.0%)	
Uveitic	1 (0.8%)	1 (1.4%)	0 (0.0%)	
**Lens State**				<0.001
Phakic	68 (59.6%)	22 (32.3%)	46 (100.0%)	
Pseudo-phakic	46 (40.3%)	46 (67.4%)	0 (0.0%)	
**Previous Surgeries/Laser**				0.2
None	97 (85.1%)	55 (80.8%)	42 (91.3%)	
Trabeculectomy	1 (0.8%)	1 (1.5%)	0 (0.0%)	
SLT/ALT	17 (14.9%)	13 (19.1%)	4 (8.7%)	
Iridotomy	4 (3.5%)	2 (2.9%)	2 (4.2%)	
MIGS	9 (7.9%)	7 (10.3%)	1 (2.1%)	
**Other**	4 (3.5%)	3 (4.4%)	1 (2.1%)	
**VF Damage**	**(n = 78)**	**(n** **= 39)**	**(n = 39)**	
MD (dB), median (IQR)	−15.15	−16.25	−13.4	0.3
	(−22.9; −5.05)	(−23.9; −6.3)	(−21.0; −4.2)	
Better than −6 dB	22 (28.2%)	10	12	0.8
−6 to −12 dB	12 (15.3%)	6	6	
Worse than −12 dB	44 (56.4%)	23	21	
**C/D Ratio,** **median (IQR)**	(n = 12) 0.7 (0.67; 0.85)	(n = 9) 0.7 (0.6; 0.8)	(n = 3) 0.85 (0.77; 0.85)	0.4
**Global RNFL (µm),** **median (IQR)**	(n = 54) 63.5 (53.2; 74.7)	(n = 31) 64.0 (53.5; 78.5)	(n = 23) 64.0 (56.0; 70.5)	0.6
**Endothelial Count,** **median (IQR)**	(n = 32) 2186 (1884; 2452)	(n = 11) 1997 (1890; 2104)	(n = 21) 2332 (1870; 3068)	0.2

**Table 2:** Complications.

	XEN63					XEN63 + Phaco				
	1D	1W	1M	3M	6M	1D	1W	1M	3M	6M
Bleb - Fibrosis - Cystic - Flat - Avascular	- - - -	- - - -	- - - -	2 - - 1	6 1 - -	- - - -	- - 1 -	- - 1 -	3 - - -	2 - - -
Choroidal bleb Choroidal detachment	1 2	- 3	- 2	- 1	- -	- 1	- -	1 -	- -	- 1
Hyphema	4	1	-	-	-	1	1	-	-	-
Iris–XEN63 contact	1	-	-	-	2	-	1	-	-	-
XEN63 Obstruction - Fibrin	- -	1 -	1 1	- -	1 -	- -	- -	1 -	- -	2 1
Hypotony - Maculopathy	1 -	- -	1 1	- -	- -	- -	- -	- -	- -	- -
Hypertension (>30 mmHg)	-	1	-	1	1	-	-	1	1	-
XEN63 - Dislocation in AC - Subconjunctival malposition	- -	- -	- -	1 1	- -	- -	- 1	- -	- 1	- 2

**Table 3:** Post-operative procedures.

	XEN63						XEN63 + Phaco					
	1D	1W	1M	2M	3M	6M	1D	1W	1M	2M	3M	6M
**Needling** - Without antimetabolites - With antimetabolites	- -	1 -	3 1	1 5	2 2	2 2	3 -	2 -	7 1	4 2	3 6	4 2
**Laser Iridoplasty**	-	1	-	-	-	-	-	1	1	-	-	-
**PhacoTrab**	-	-	1	-	-	3	-	-	-	-	-	-
**Trab**	-	-	-	-	-	3	-	-	-	-	-	1
**PreserFLo**	-	-	-	-	1	-	-	-	-	-	-	-
**XEN ab externo**	-	-	-	-	1	1	-	-	-	1	-	-
**Surgical revision**	-	-	1	-	1	2	-	-	1	2	-	1
**Anterior Chamber refill**	-	-	-	1	-	-	-	-	-	-	-	-

## Data Availability

The data presented in this study are available on request from the corresponding author. The data are not publicly available due to privacy restrictions.

## Abbreviations

The following abbreviations are used in this manuscript:

AC	anterior chamber
IOP	intraocular pressure
MMC	mitomycin C
OHMs	ocular hypotensive medications
POAG	primary open-angle glaucoma
